# Recommendations for a Communication Strategy to Support Informed Decision‐Making About Self or Clinician Sampling for Cervical Screening in the UK: Qualitative Study

**DOI:** 10.1111/hex.70191

**Published:** 2025-03-27

**Authors:** Denitza Williams, Eleanor Clarke, Kate J. Lifford, Lindsay Haywood, Fiona Wood, Jo Waller, Adrian Edwards, Natalie Joseph‐Williams, Caroline Evans, Gareth Powell, Rhiannon Phillips, Andrew Carson‐Stevens, Katie Walbeoff, Ardiana Gjini, Kate Brain

**Affiliations:** ^1^ Division of Population Medicine, School of Medicine, College of Biomedical and Life Sciences Cardiff University and PRIME Centre Wales Cardiff UK; ^2^ Public Partner UK; ^3^ Centre of Cancer Screening, Prevention and Early Diagnosis, Wolfson Institute of Population Health Queen Mary University of London London UK; ^4^ PHW Screening Laboratory, Public Health Wales Cardiff UK; ^5^ Cardiff School of Sport and Health Sciences Cardiff Metropolitan University Cardiff UK; ^6^ Cervical Screening Wales, Public Health Wales Cardiff UK; ^7^ Hywel Dda Health Board, NHS Wales UK

**Keywords:** behaviour change, cervical screening, communication, decision support, human pappilomavirus, self‐sampling

## Abstract

**Background:**

Cervical screening for high‐risk Human Papillomavirus subtypes is offered to those eligible in the UK via the NHS cervical screening programmes. However, uptake of cervical screening continues to remain below the national target of 80%. Groups less likely to participate include people from low socioeconomic groups, ethnic minority backgrounds, younger/older age and/or LGBTQ group identity. The cervical screening‐eligible population could soon, for the first time in the UK, have a choice of mode between clinician taken or self‐sampling.

**Aims:**

To understand information and decision‐support needs of diverse cervical screening‐eligible individuals when presented with a choice of cervical screening mode and develop recommendations for a communication strategy to support informed decision‐making.

**Methods:**

Qualitative co‐production explored communication preferences and decision‐support needs in a diverse sample of cervical screening‐eligible individuals using semi‐structured interviews with individuals eligible for cervical screening (*n* = 30) and stakeholders (*n* = 23). Interviews were transcribed, thematically analysed and mapped to behavioural and decision‐making theories to inform a communication strategy for offering choice in cervical screening mode in the UK.

**Results:**

Four main themes across both participant groups were identified: misunderstanding of clinician screening, attitudes towards choice, communication launch preferences and decision‐support needs. Logic models to inform a communication strategy in preparation for the future launch of choice in cervical screening mode in the UK were developed.

**Implications:**

The communication launch strategy can inform interventions to support informed decision‐making if HPV self‐sampling is incorporated into UK cervical screening programmes.

**Patient and Public Contribution:**

Two public partners were involved in the study from inception to completion. They advised on recruitment, participant facing documents and were involved in analysis.

## 1 Introduction

Cervical cancer is the second most diagnosed cancer in individuals eligible for cervical screening under 45 years of age in the UK [1]. Clinician screening using high risk human papillomavirus (HPV) testing is routinely offered to women and other individuals with a cervix between the ages of 25 and 64 years via the NHS cancer screening programmes in the UK [2, 3, 4, 5]. Cervical screening coverage has steadily declined in the UK over the last decade and currently stands at 69.6% in Wales [6], 68.7% in Scotland [7], 68.8% in England [8] and 66.7% in Northern Ireland [9]. Uptake remains below the target level of 80% which is needed to reduce incidence of cervical cancer and to ensure screening cost‐effectiveness [10].

The HPV vaccination programme was introduced in the UK for under 18s in 2008, with potential to prevent over 80% of the most common types of cervical cancer [11]. However, it could be decades until the UK population comprises mainly of vaccinated individuals and screening remains the main strategy for cervical cancer prevention. Individuals from low socioeconomic groups [12], ethnic minority backgrounds [13], younger/older age groups [13, 14, 15], those who have disabilities [16], transgender men and nonbinary people [17] and those who are lesbian and bisexual [18] are less likely to participate in cervical screening. During the COVID‐19 pandemic, cervical screening was paused temporarily [19]. When screening re‐started in the UK, one in five eligible individuals reported being less likely to take part [20]. Common barriers [21, 22] to cervical screening such as embarrassment, body image concerns, lack of available appointments, cultural sensitivity or time to attend [21, 22], could be reduced by providing the option of HPV self‐sampling, where a sample for HPV testing is taken by the person themselves at home or other preferred location. Taking sampling out of the clinic setting could also reduce workload pressures on primary care staff.

The European Cancer Organisation recommends HPV self‐sampling as a central component of organised cervical screening programmes [23]. HPV self‐sampling could have a positive impact on uptake in under‐screened populations [24, 25]. HPV self‐sampling has already been implemented in several countries such as Australia and Denmark and the WHO has recommended that HPV self‐sampling is an additional approach to sampling in cervical screening [26]. In 2019, HPV self‐sampling was welcomed as a potential option for cervical screening in a UK joint position statement [27]. The HPValidate study in England identified that while some self‐sampling methods were effective [25], participants welcomed the choice in cervical screening mode but needed support selecting HPV self‐sampling or clinician sampling [28]. The UK National Screening Committee has called for research into the acceptability and clinical feasibility of HPV self‐sampling screening and patients' preferred involvement in decisions about cervical screening mode [29].

**Figure 1:**
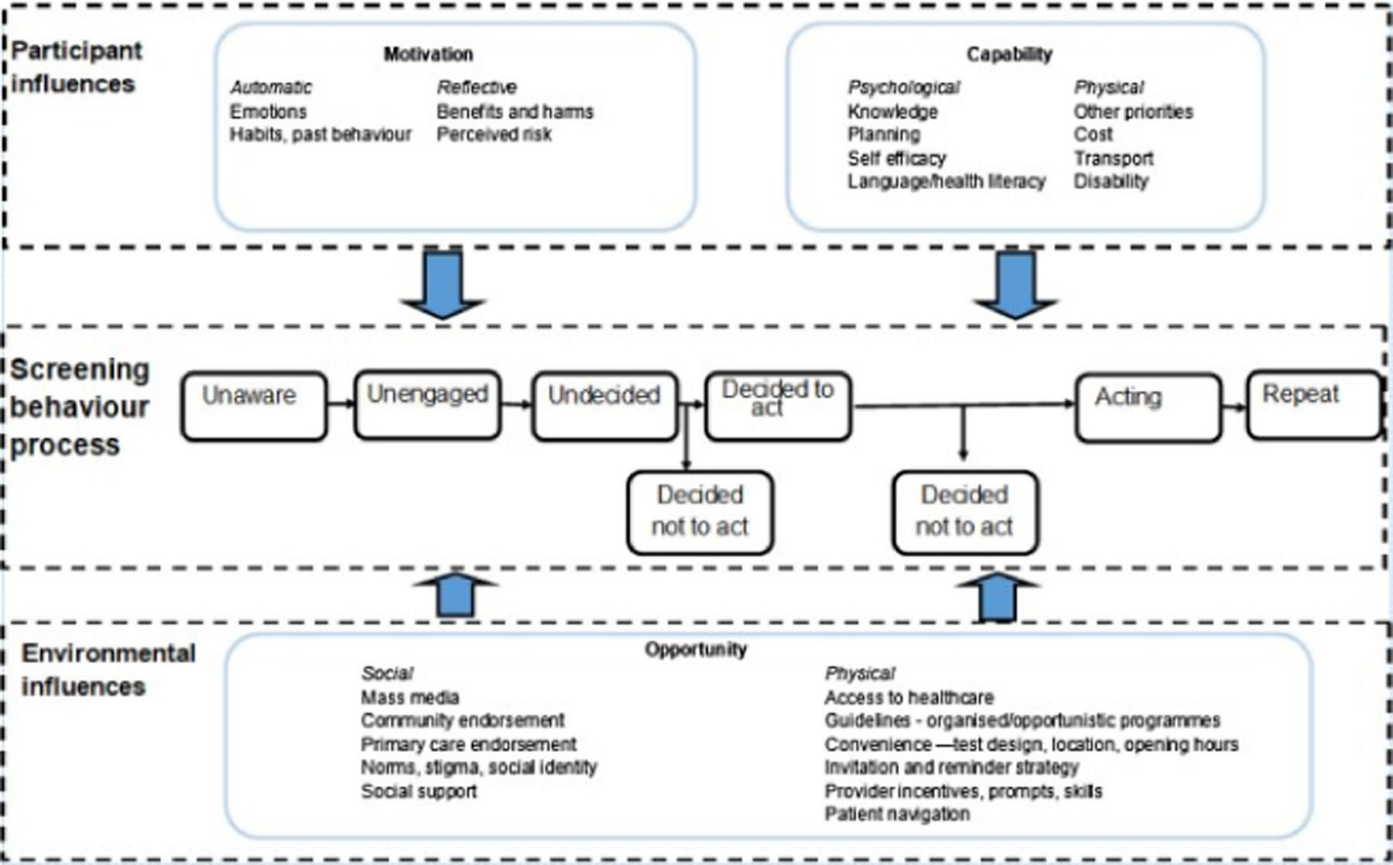
Integrated screening action model (I‐SAM). Replicated with permission from Robb (2019). https://doi.org/10.1016/j.pmedr.2021.101427.

HPV self‐sampling can address some of the barriers to traditional clinician sample collection. It also presents unique barriers, including low confidence in personal ability to collect a sample and lack of trust in the result [30, 31]. UK healthcare policy advocates preference‐based decision‐making through the concept of shared decision‐making when choices exist [32]. Informed decision‐making is a process that enables individuals to make health‐care decisions after they have considered information about the options available and how options fit with their preferences [33]. If HPV self‐sampling becomes an additional option for cervical screening in the UK, it will be important to incorporate decision support into cervical screening programmes to enable informed decision‐making at population level, and to ensure that the offer of choice does not have a negative impact on engagement in under‐screened populations [33]. The way in which changes to the cervical screening programmes are communicated to the public is important to avoid negative public perceptions and lack of trust [34, 35]. Theoretical models such as the integrated screening action model (I‐SAM) [36] and Implement‐SDM [37] can be used to understand behaviour and guide targets for interventions [38], such as communication strategies to promote informed decision‐making [33] about choice in cervical screening (self‐sampling vs clinician sampling). As depicted in Figure 1, the I‐SAM expands the Precaution Adoption Process Model (PAPM) [39] by conceptualising screening participation behaviour as a pathway from individuals being unaware of and unengaged with screening options, to deciding whether or not to take part in screening and adhering to repeat screening rounds. The individual's perceived capability, opportunity and motivation also influence their movement across the screening pathway. In cervical screening, the I‐SAM can incorporate a dynamic screening communication and decision pathway for individuals who are at various stages of the decision‐making process. The I‐SAM is particularly complemented by the Implement‐SDM model which outlines the stages needed to facilitate informed decision‐making through a process of shared decision‐making.

**Figure 2:**
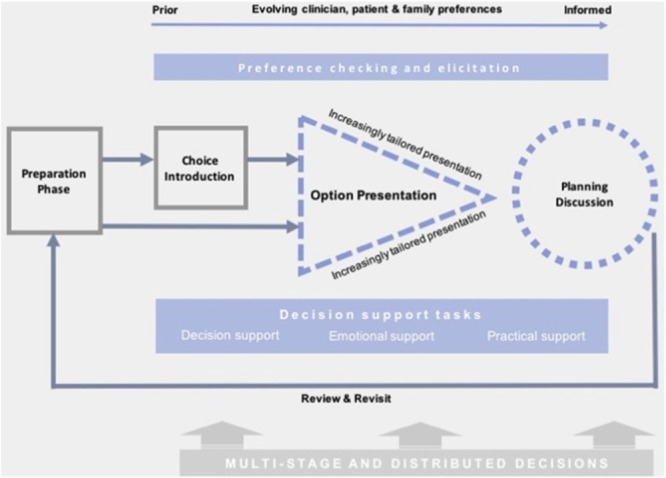
‘Implement‐SDM’ – Descriptive model of shared decision‐making based on observations of routine practice. Replicated with permission from Joseph‐Williams et al. (2019). https://doi.org/10.1016/j.pec.2019.07.016.

Implement‐SDM (Figure 2) interjects at the ‘undecided’ stage of the I‐SAM screening behaviour process, with potential to support deliberation and preference‐based decision‐making for informed cervical screening participation (including repeat participation). Furthermore, Implement‐SDM is able to account for the distributed nature of the decision‐making process over initial and repeat cervical screening rounds.

In the SUCCEED study, we aimed to develop evidence‐based recommendations for a communication strategy to support informed decision‐making for choice between HPV self‐sampling or clinician HPV sampling, should HPV self‐screening become available in the UK. The study was underpinned by the I‐SAM [36] and Implement‐SDM [37] models.

## 2 Methods

### 2.1 Study Design

The SUCCEED study involved qualitative semi‐structured virtual interviews with individuals eligible for cervical screening and key stakeholders. Logic models were used to visualise our emergent theory of how the proposed communication strategy could produce outcomes of interest [40].

### 2.2 Sample and Recruitment

#### 2.2.1 Eligibility Criteria

##### 2.2.1.1 Screening‐Eligible Sample

Inclusion criteria: eligible for UK cervical screening (aged 25–64 years of age with a cervix).

Exclusion criteria: unable to read/speak English.

##### 2.2.1.2 Stakeholder Sample

Inclusion criteria: professional working within a relevant organisation including third sector, community organisations and professional stakeholders (e.g. GPs, sexual health doctors, speciality doctors, practice nurses, staff from Public Health agencies, charities).

Exclusion criteria: unable to read/speak English.

### 2.3 Recruitment

Individuals eligible for cervical screening were purposively sampled for diversity in screening participation, age, education level, ethnicity and LGBTQ status and were recruited by a market research company, Taylor McKenzie (T.M.). T.M. were used for recruitment because they have a track‐record of being able to recruit underrepresented populations within research and were able to recruit participants for healthcare studies for maximum variation. They have national databases as well as local community hubs to facilitate diverse recruitment of individuals. Stakeholders were recruited by direct e‐mail, professional contacts and e‐mail distributions to relevant networks.

Recruitment for interviews was guided by the concept of ‘information power’ [41] and continued until no significant new themes were identified [41].

### 2.4 Interview Procedures

Participants were sent an information sheet and consent form at least one week before the interview. Interviews were conducted remotely via Zoom/Teams. Verbal consent was audio‐recorded at the start of the interview.

Interviews were guided using semi‐structured interview schedules (Appendix 1) that were informed by I‐SAM [36] and Implement‐SDM [37]. The interview schedule for eligible individuals included topics focusing on how best to communicate a new choice in cervical screening mode, communication needs, screening intentions, attitudes towards choice in cervical screening, decision‐support needs and communication preferences.

The interview schedule for stakeholders included barriers and facilitators to implementation of HPV self‐sampling, and the development and implementation of a communication strategy to support informed screening participation.

All interviews were digitally audio‐recorded and transcribed verbatim. One researcher, E.C., conducted all interviews. E.C. is an experienced qualitative researcher who identifies as a white female and is eligible for cervical screening.

### 2.5 Data Analysis

Interview transcripts were thematically analysed [42], supported by NVIVO version 1.7.1 software [43]. Thematic analysis consisted of data familiarisation, generation of initial codes, searching for themes, reviewing and refining themes, and defining and naming final themes [42]. Analysis was primarily conducted by E.C. and D.W. To maximise rigour and reflexivity, regular team meetings including public partners (attended by D.W., E.C., L.H. and C.E.) were used to discuss development and application of the coding framework and data analysis. Interviews with screening‐eligible individuals and stakeholders were analysed separately.

### 2.6 Logic Model Development

Logic models were used to visualise how the proposed communication strategy could produce the desired outcome of informed decision‐making about cervical screening [32]. Data were triangulated from individuals and stakeholders to facilitate a multidimensional understanding [36] of behaviour change and decision‐support needs. Interview themes were mapped to the I‐SAM [36] and Implement‐SDM [37] models to develop logic models for communication of cervical programme changes to support informed decision‐making. I‐SAM was used as a screening behaviour process model outlining how decision‐making behaviours vary according to the individuals' awareness/knowledge and engagement throughout the screening pathway. Implement‐SDM was used to define the specific processes needed to facilitate informed decision‐making (such as choice introduction, outlining pros and cons of each sampling mode and preference elicitation) as individuals progress through the I‐SAM pathway. Themes were allocated to distinct categories within the models which were input, activities, outputs and outcomes.

### 2.7 Patient and Public Involvement

Public involvement in the study was underpinned by the UK Public Involvement Standards [44]. Three public involvement partners were part of the core study team involved in applying for funding and full development of this study. Two of the members (L.H. and C.E.) continued to be involved throughout the execution of the study, contributing to participant documentation, interview schedules, recruitment adverts, analysis workshops (including reviewing transcripts, analysing data and informing the development of themes), dissemination through conference attendance and co‐authoring this manuscript.

### 2.8 Ethical Considerations

The study was approved by the School of Medicine Research Ethics Committee at Cardiff University (SMREC 22/88). Interviews explored a potentially sensitive topic. Overall, we appraised the risk of harm as low but recognised potential for some participants to become upset during the interview if recalling negative medical experiences. The participant information sheet contained contact details of relevant sources of support (Jo's Cervical Cancer Trust, Mind, Samaritans).

## 3 Results

Throughout the interactive recruitment process, recruitment stopped once no new significant themes were identified during the interviews which was 30 interviews. Throughout the process thirty‐five individuals eligible for cervical screening expressed an interest in taking part in an interview; thirty individuals were interviewed (86%) (Table 1), Five out of the thirty‐five individuals did not participate for the following reasons: change in personal circumstances (*n* = 2), withdrew consent (*n* = 1), lost contact (*n* = 1), ineligible (*n* = 1). Participants had a wide range of demographic characteristics. This included age (mean 37.9 years, range 25–63, standard deviation 10.52), screening history (never screened *n* = 10, late or irregular responder *n* = 7, regular responder *n* = 13) and ethnicity (White/White British *n* = 16, other ethnic groups *n* = 14). Most participants identified as female (*n* = 28) and most were educated below degree level (*n* = 15). Interviews lasted an average of 37 min (range 20–53 min).

**Table 1:** Screening‐eligible participant demographics.

		Phase one participants, *n*			
		Never Screened, *n*	Late or irregular cervical screening responder^a^ *n*	Regular cervical screening responder, *n*	All, *n*
Total in response category		10	7	13	30
**Self‐identified**	Female	9	7	12	28
**Gender**	Male	1	0	0	1
	Nonbinary	0	0	1	1
**Age (years)**	24–33 years	7	0	5	12
	34–43 years	2	3	6	11
	44–53 years	1	2	1	4
	54–64 years	0	2	1	3
**Time since last cervical screening (years)**	0–3 years	0	0	10	10
	4–5 years	0	2	2	4
	5–10 years	0	3	0	3
	10 years or more	0	2	0	2
	Never	10	0	1^a^	11
**Highest level of education**	School or college (incl. GCSE)	1	4	5	10
	A level	0	1	2	3
	NVQ	1	0	0	1
	Diploma	1	0	0	1
	Degree or university level	6	2	6	14
	Unknown	1	0	0	1
**Sexual orientation**	Heterosexual	9	6	10	25
	Other	1	1	3	5
**Nation**	England	7	5	7	19
	Wales	0	0	4	4
	Scotland	3	2	2	7
**Self‐described ethnicity**	White	1	1	3	5
	White British	2	4	5	11
	Indian	1	1	1	3
	Pakistani	0	1	0	1
	British Pakistani	0	0	1	1
	Greek Cypriot	1	0	0	1
	Asian	1	0	0	1
	African	1	0	0	1
	Black African	1	0	0	1
	Black Caribbean	1	0	0	1
	Asian and white	0	0	1	1
	Mixed black and white British	0	0	1	1
	Mixed Asian and white	1	0	0	1
	Mixed race	0	0	1	1

^a^ Late/irregular cervical screening responder defined as more than five years since last cervical screen (or more than 3 for some in England and Scotland) and having ever screened.

Twenty‐three stakeholders were recruited, from a range of backgrounds; *n* = 12 clinical and public health, *n* = 9 third sector organisations, *n* = 2 mixed clinical and third sector organisations (Table 2). Stakeholder interviews lasted an average of 36 min (range 16–53 min).

**Table 2:** Stakeholder roles and nations.

	Stakeholder, *n*
**Stakeholder Role**	
**Clinical**	**10**
GP	3
Nurse	5
Public Health consultant	1
Sexual Health consultant	1
**Public Health**	**2**
**Third Sector**	**9**
Patient‐led charity	2^a^
LGBTQ charity	1
Cervical cancer charity	1
Domestic and/or sexual abuse charity	2
Community champion charity	2^a^
Faith organisation	1
**Mixed Role:** Clinical and third sector	**2**
**Stakeholder nation of work**	
All nations	1
England and Wales	3
England	7
Wales	8
Scotland	4
All	23

^a^ Participants who participated in interviews in a pair.

Four main themes across both participant groups were identified: misunderstanding of clinician screening, attitudes towards choice, communication preferences and decision‐support needs. Each theme included multiple sub‐themes, which will be described below with exemplar quotes, with participants referred to as SH = stakeholder and where their role is based (e.g. SH23, public health) and P = individuals eligible for cervical screening, their cervical screening attendance and gender identity (e.g. P13, nonresponder, male).

### 3.1 Misunderstanding of Clinician Screening

#### 3.1.1 High Perceived Knowledge

Individuals reported high perceived knowledge about cervical screening. However, their knowledge was often inaccurate, particularly in relation to the shift to HPV primary screening in the UK. This specific gap in knowledge amongst the screening‐eligible population was echoed by stakeholders. Individuals generally believed that all cervical screening still involved investigation of cellular changes at the outset instead of HPV presence (consistent with previous primary cytological screening). Some thought it was a more general gynaecological check‐up, beyond HPV. This knowledge was often gained generationally or through lay networks.

I know a lot of cis‐females who are needing to have it [cervical screening] done but they're younger […] they keep asking the doctor and they're like 23, 24 so it's not […]they have problems with their periods and stuff […] My missus did the exact same. She wanted one ever since she was 18. And they were like ‘No’.

P13, non‐responder, male

I think a lot of knowledge, in my experience, from talking to people, has come from mothers.

SH23, Public Health

Individuals reported being fairly unengaged in cervical screening educational opportunities.

I knew the, the basics of it, I just didn't really look into it [information] because I had no need to.

P14, regular attender, non‐binary

The misunderstanding of current clinician sampling seemed to have a direct impact on the level of trust associated with an HPV self‐sampling offer which led to problems when comparing the two types of sampling during the interview.

it feels like it might be an inferior test […] So, if this swab isn't going to the cervix, it's not going to detect cervical cancer. It's detecting HPV, which is that… STD?

P20, late or irregular attender, female

Stakeholders also reflected on a lack of knowledge displayed by patients or clients, with some clinical participants reflecting on a lack of personal understanding before their own clinical training. Stakeholders reflected that individuals misunderstood not only the method of cervical screening but also the results that were presented.

this routine screen, a lot of people are not understanding what their own results mean.

SH16, nurse

### 3.2 Attitudes Towards Choice in Cervical Screening Mode in the UK Programmes

Overall, both individuals and stakeholders were positive about supporting choices in healthcare and most felt that offering a choice in cervical screening mode was a positive step within the UK cervical screening programmes.

I just think that when you're trying to reclaim back your life, it's nice to make your own decisions […] I just know it is lovely to have a choice and have that choice validated.

SH10, third sector organisation

It's always best to kind of give people an option, then they don't feel like I'm being forced to do something here.

P11, never screened, female

However, a minority of individuals felt that the cervical screening service is adequate and thus felt that there is no need to introduce a change such as this.

Um, so, if it's somebody that's doing it all the time, like myself, then I'm like, well, don't change it, you know, if it's not broke, don't fix it.

P4, regular attender, female

Altruistic perspectives were shared by most individuals who didn't see HPV self‐sampling as helpful for themselves. They often reflected that self‐sampling could remove some barriers associated with clinician sampling and could support others less likely to engage in clinician sampling.

I think it's … yeah, definitely, good idea to have a choice (…) it opens that up for people that maybe are like me, or, people that are maybe a bit uncomfortable to do the screening, you know, they would prefer to do it in their own home, you know, in the privacy of their own home. Um, and maybe they trust themselves more. I know a lot of people, um, have said like they don't trust doctors.

P14 regular responder, non‐binary

#### 3.2.1 Financial Motivation

Some were sceptical about the introduction of a self‐sampling choice. This involved concern that self‐sampling may be offered to save the NHS money at the expense of health outcomes. Clinician stakeholders expressed concerns that patients could perceive self‐sampling as cost‐cutting rather than a legitimate and positive step toward person‐centred options in screening. Some cervical screening‐eligible individuals and stakeholders also felt that the offer of self‐sampling would lead to a lack of opportunity to engage with a healthcare professional about gynaecological issues.

It's just a cost cutting exercise I think (…) Well, yes, I do. I mean everything, I'm very, very cynical.

P5, regular attender, female

However, the cost‐saving nature of self‐sampling was not seen as negative by all participants and some perceived a financial saving as a benefit of self‐sampling for the NHS.

I think yeah, we're all trying, if anything, it's cheaper and yeah, cost effective, that's a good thing, I would imagine.

P33, late or irregular attender, female

#### 3.2.2 Trust in Ongoing Authentic Choice

Screening‐eligible participants wanted reassurance they would have a genuine choice and could change their mind in relation to mode of cervical screening during this and subsequent screening cycles should they wish. The concept that self‐sampling may become the only mode available for cervical screening, to save resources, was raised as a concern (or assumption) by some participants. This suggests a lack of trust for the motivation behind introducing choice.

I like to have flex, when I'm making decisions, I don't like to be put on the spot. This is, like, because then I think oh, fuck that then. But if I've got a choice, I'm more inclined to sit there, and think about it.

P21, non‐responder, female

They kind of need to know that it's their choice, their body, their choice.

P6 non‐responder, female

in terms of whether there should be an option? I mean, I guess, while it's [cervical screening] being phased out.

SH22, mixed role

Other cervical screening‐eligible individuals were concerned that once they had made a choice in cervical screening mode that they would not be offered a choice again. For example, some participants reflected on the introduction of self‐sampling as a potential quick fix for access to groups of individuals who are less well catered for in clinical settings, such as wheelchair users. There was concern raised that these groups would then only be offered HPV self‐sampling and not clinician sampling in the future as a quick fix for access issues, potentially increasing inequalities.

#### 3.2.3 Test Accuracy

There was concern from cervical screening‐eligible participants about their own and others' ability to conduct self‐sampling. There was also concern that even if it was done correctly that self‐sampling would not be as accurate as clinician sampling and could lead to false negatives.

how high you need to go or, do you know what I mean, I think, are you doing it correctly? I'd be worried, err, it coming back, and they'd it, it'd be a false reading, cos I've not done it properly.

P15, regular responder, female

### 3.3 Communication Preferences for Introduction of Change in Cervical Screening Programmes

The importance of a clear multi‐modal and multi‐agency communication strategy was identified, a lead‐in period for communication of upcoming changes was seen as imperative.

you'd like to think there would be a, a national campaign […] belts and braces, I guess.

P33, late or irregular responder, female

If we're gonna bring in self‐sampling, I think it needs to be accompanied by a huge education campaign. I think what we've got is probably twenty years messaging from, not even messaging, from the programme, but twenty years of kind of, societal knowledge around the subject.

SH11, Public health

#### 3.3.1 Preparation Phase

Preparation for change was seen by both screening‐eligible participants and stakeholders as a critical first step in communication about the introduction of choice in cervical screening. This also highlighted the need to signal to the public that cervical screening has already changed (to challenge misunderstanding and re‐engage interest). Perceived length of the preparation phase varied from weeks to over 12 months.

for women who are turning up for smear tests, in the next twelve months, 2 years, are also then being given something, that says, you know, next time you come, you will have options, you can do it like this. Then, whilst they're doing what they're doing, you know, they, they're talking about the options, then that's a way of getting that across.

SH15, Third sector

I think, in advance would be good so that people are prepared for it. So, they get like maybe a letter a couple of months before they're due, like explaining what it is and what it does, and how, how it compares.

P19, late or irregular responder, female

The trusted source and author/narrator of the communication launch was seen to be the ‘NHS’ overall, as opposed to other sources such as public health or third sector organisations. Participants reflected that a multiplatform and multichannel approach will be needed to ensure equitable reach that appeared trustworthy and credible.

#### 3.3.2 Transparency

Some participants were cynical of the agenda of government agencies (see financial motivation subtheme), held a disbelief that self‐sampling could be a simple process and that it is something that has only recently been possible as an offer. Some participants wanted access to more detailed information, through links and/or referencing.

if it was as simple as that, literally just putting it in, turning it and bringing it back out, then how come, you know, it's only now that it's kind of being introduced, basically why has it taken so long if it was as simple as that.

P11 non‐responder, female

#### 3.3.3 Visual Risk Communication Aids

Participants discussed different needs in understanding risk and test comparisons in relation to efficacy to address low health literacy and low numeracy. Some wanted visual aids such as icon array for risk communication. Multichannel preferences were identified for accessing information about cervical screening including videos. Echoing the positivity around choice in healthcare, people wanted choice in how they could access information.

#### 3.3.4 Cultural Sensitivity

Some participants were comfortable with anatomical and gendered terms around cervical screening. There was some discussion that people need to be better informed about their health and bodies, and that ‘*proper terminology*’ (P18) should be used for clarity. However, other participants urged caution with imagery and words so as not to isolate groups, such as religious and LGBTQ communities.

something that I think would be useful is to not necessarily gender it a lot. Um, and, yeah, keeping it quite neutral. Just, yeah, anyone with a cervix needs to get a screening.

P8, regular attender, female

with the other people then it'll be really strange to talk about self‐sampling, I don't really think, well perhaps they could make it a little bit more comfortable by referring to things that's not too obvious, you know, rather than call it vagina you can just say private area, you know, as an example.

P34, never screened, female

### 3.4 Decision‐Support Needs

Cervical screening‐eligible participants expressed that they would need support to make an informed decision about mode of HPV screening. There was a perceived need for a decision aid that would aid informed decision‐making.

we don't know what decision people are making, because it's not recorded. So, it would be interesting to know if people are making a decision, not an informed decision, that is, not to participate, because of cultural, err, religious reasons or whatever, because then I think there's very much a focus needs to be done, of the what next?

SH17, Public Health

Preferences for format of a decision aid were variable but included online website, app and paper‐based booklet within the screening invite. Individuals spoke about a varying need for depth of information through multiple versions of a decision aid.

Participants spoke of key content that would be needed within a decision aid. These are described in the following sub themes.

#### 3.4.1 How Screening Works?

Some cervical screening‐eligible participants were unclear about how cervical screening works and their eligibility for cervical screening (asymptomatic presentation). Some assumed that they would be able to order an HPV self‐sampling kit for gynaecological symptoms or that they (or their relatives) should not be screened if they were HPV vaccinated. Therefore, there was a high need for eligibility information to be clearly presented.

I would want to know what are the symptoms to look out for. And, when is it that I should feel the need to test, basically.

P28 non‐responder, female

Maybe every 5 years, I can go to my GP, but in the middle of that, can I do a self‐test, just to make sure that everything still looks okay?

P1 responder, female

does the NHS still recommend you do it, even if you have the vaccine? And if that's true (…) then I think that should definitely be on it. People will think, oh I have the HPV vaccine, I don't have to do it.

P25 non‐responder, female

#### 3.4.2 False‐Negative Results

Cervical screening‐eligible participants wanted information about the likelihood of a false‐negative HPV self‐sampling test result. Comparison of the performance of the self‐test with clinician screening was important as it was assumed clinician testing would be superior by many of the cervical screening‐eligible participants. Participants suggested a number of different ways to present the comparison of test performance. Perceived self‐efficacy in completing self‐sampling adequately was also closely related to the perception that specialist skills are needed for HPV sampling.

I'd like to compare percentages from the one in the clinic. Compared to the one you do at home. Because I don't want to be doing one at home if I don't know, it's coming back again […] Let's say the virus was there and I'm not all the way up to the, wherever I need to be or whatever, I'd just keep doing this. Wherever I am, what is the difference between you and me doing it?

P13 non‐responder, male

You know, if you get the information that says, if you come and do it with us, there's a ninety five percent chance that it's accurate, but if you do it yourself, it's a seventy percent chance it's accurate, or whatever, you know.

P1 responder, female

#### 3.4.3 Onward Cervical Screening Journey

Participants reported a need to fully understand the onward cervical screening journey after HPV self‐sampling for those who test HPV positive (attending for a clinician sample). For some participants, this was crucial information in their decision‐making. Some non‐attenders felt that the need for subsequent clinician testing would present a barrier to engaging with self‐sampling. Other non‐attenders felt that an HPV‐positive result through self‐sampling would justify the need for subsequent clinician sampling.

I think it would encourage more people, like me, who are sort of a bit nervous about it, like perhaps it would like, bridge the gap, I'm sure there's some people who still wouldn't but.

P30 non‐responder, female

Participants also discussed a need to understand what results may mean, what format the results would come in (e.g. letter, text message), how long the results would take and how individuals would be supported through the screening programme if needed.

#### 3.4.4 How to Self‐Sample?

Cervical screening‐eligible participants felt that they would need key information about the HPV self‐sampling procedure in a decision aid to help inform their preferences. Key pieces of information included not needing to reach the cervix, whether the swab devices will have a marker to indicate the depth of insertion, frequency of self‐sampling and continual support for informed decision‐making at each screening round.

#### 3.4.5 Pathway Navigation

Some participants felt that further support to help inform a preference‐based decision about choice of sampling mode may be of use to some, or that healthcare professionals could be approached to talk through the options. Some felt this could be a helpline or online chatbot. Stakeholders also acknowledged the potential need for a helpline to accompany a decision‐ support intervention, however there was feeling it may not be busy enough to warrant the development of a full‐time position and suggestion that staffing a helpline could be incorporated within current workloads for screening nurses for example.

I think giving them that option again, is like, if you need more information, err, ring the practice, speak to a nurse, or if they want more information, just have a website. So, somebody can look at it, and, then they can decide themselves then, if that's the route they want to take.

P15, regular responder, female

#### 3.4.6 Under‐Served Groups

When discussing making a decision between cervical screening mode there was discussion of previous healthcare and screening experiences influencing individual preferences. Participants also discussed cultural background, sexual experience, trauma, ethnicity and rurality as affecting decisions about cervical screening and its mode. These were seen as important aspects to raise in a culturally sensitive decision aid. The potential need for multiple decision aids tailored for different groups was raised.

…gendered body parts, for a lot of people who are transmasculine or nonbinary, but assigned female at birth, it can be a real trigger for dysphoria, because it, kind of, is a reminder that um, that those body parts exist and that you know, those are, kind of, maybe body parts that people would rather not have. And, yeah, so, it could be quite a distressing experience, to be reminded of that.

SH2, Third‐sector

### 3.5 Logic Model Development

Qualitative data themes were triangulated and mapped to the I‐SAM and Implement‐SDM theories. During the theme mapping, it became evident that the communication strategy would need to have two distinct points. The first was the lead up to the introduction of choice in cervical screening mode which would address the unaware/unengaged stages of the I‐SAM and the unaware/choice introduction stages of the Implement‐SDM model. The second was supporting the consideration of options during the ‘undecided’ stage of the I‐SAM by incorporating the stages of ‘option presentation’, ‘increasingly tailored information’ and ‘decision‐support tools’ aspects from Implement‐SDM. This would support movement to ‘act’ phases of I‐SAM to facilitate informed decision‐making about mode of cervical screening (or decision not to screen).

**Figure 3:**
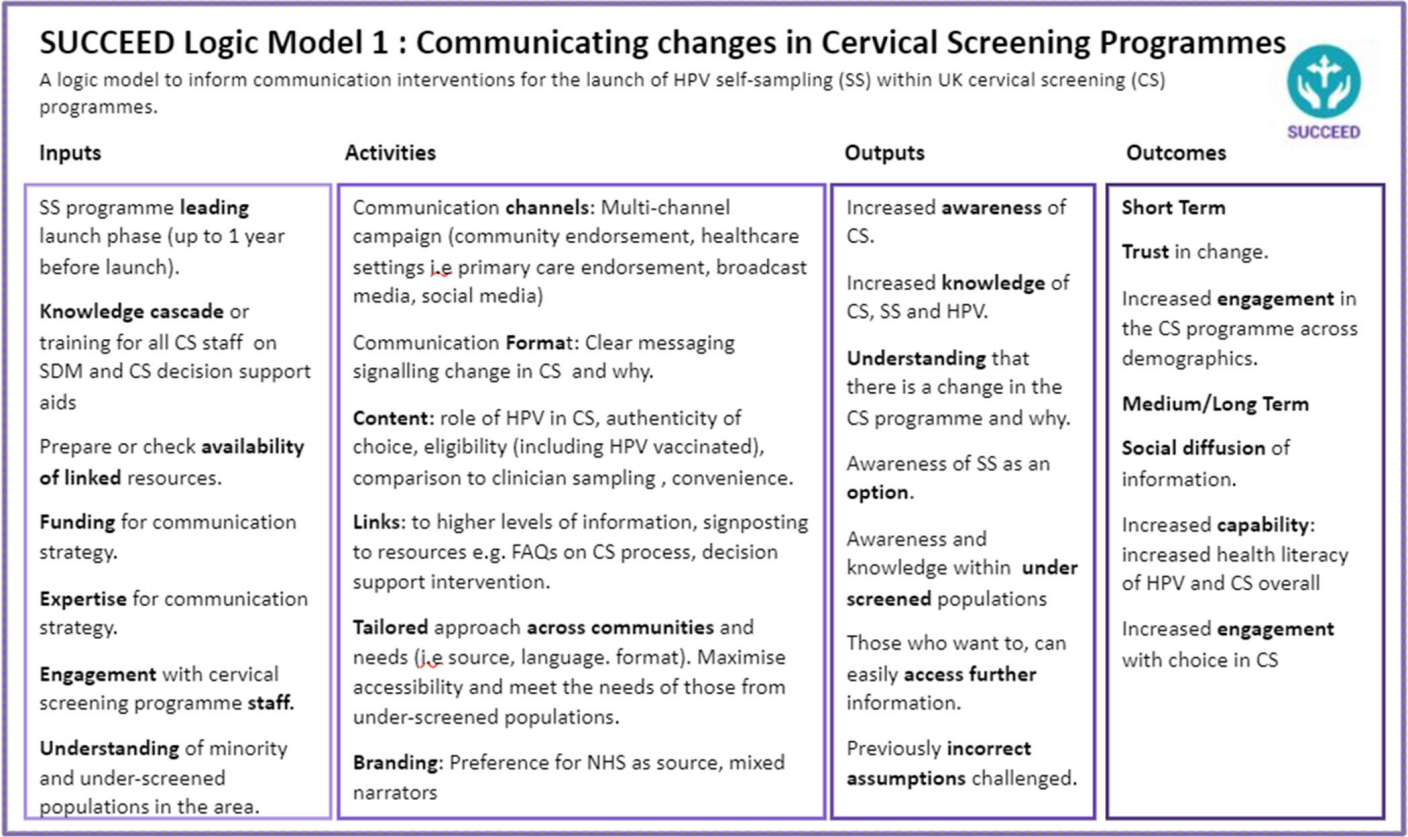
Communication strategy for launch of HPV self‐sampling in UK cervical screening programmes. Informed by qualitative findings and the I‐SAM and IMPLEMENT‐SDM models. CS = cervical screening, DA = decision aid, HPV = human papillomavirus.

**Figure 4:**
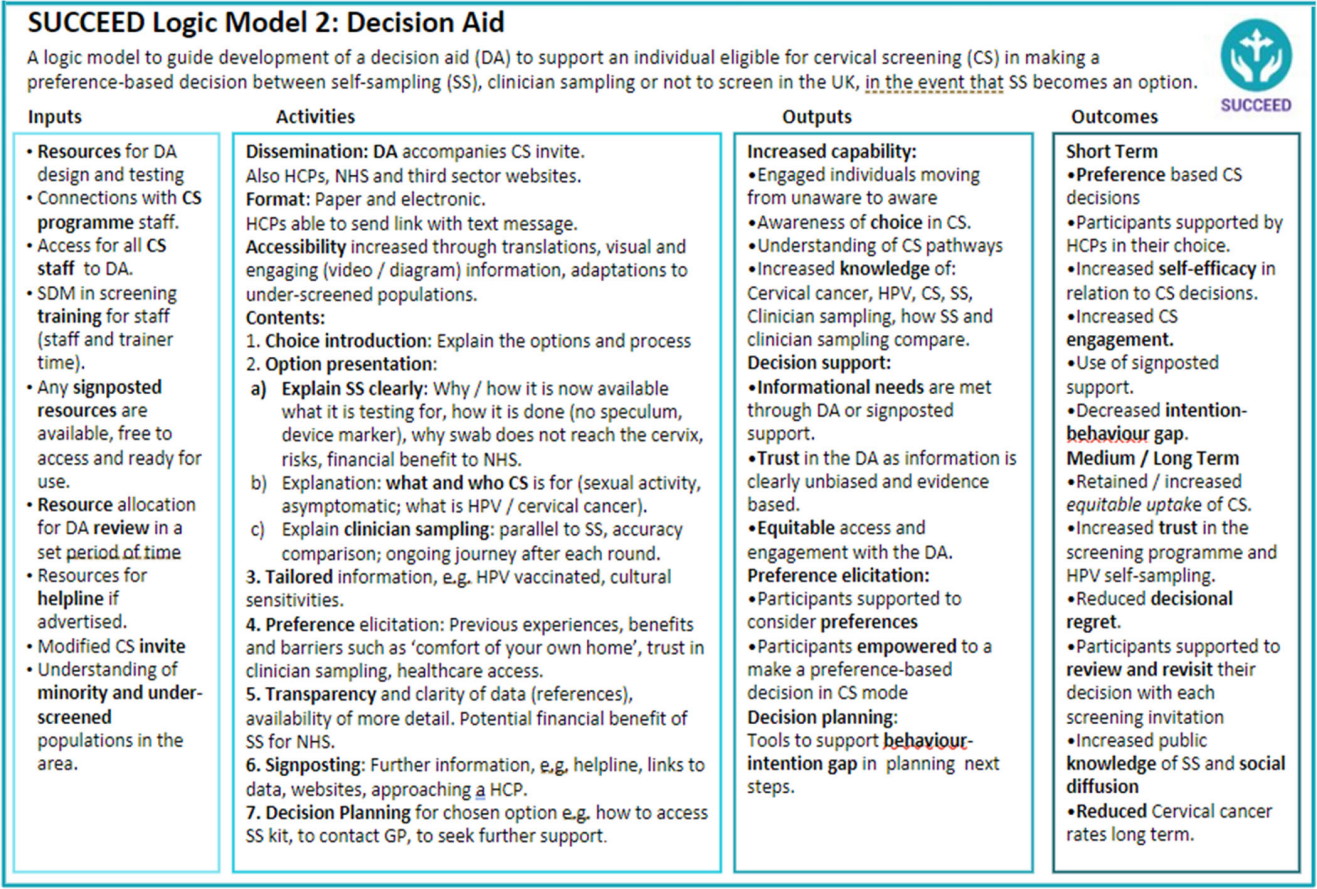
Logic Model for the development of decision aid to support equitable and person‐centred choice in cervical screening based on qualitative findings and I‐SAM and IMPLEMENT‐SDM models. CS = cervical screening, DA = decision aid, HPV = human papillomavirus.

The communication strategy is presented in two logic models, presenting theoretically informed (by the I‐SAM and Implement‐SDM) ‘active components’ needed to develop a communication strategy for offering choice in cervical screening. Model 1, the communication launch strategy model (Figure 3) targets the ‘uninformed/unaware’ aspects of choice in as per I‐SAM and the ‘preparation/choice introduction’ aspects of Implement‐SDM. The decision‐support intervention logic model, Model 2 (Figure 4) targets the ‘undecided’ aspect of I‐SAM and the ‘review/revisit’ aspects of Implement‐SDM. Both models aim to support informed decision‐making.

## 4 Discussion

There is increasing global interest in the possibility of offering HPV self‐sampling for cervical screening at population level as well as to non‐attenders, and 17 countries have already introduced self‐sampling for non‐attenders (e.g. Australia, Denmark) or as a population‐based screening option (e.g. Albania) [45]. The World Health Organization endorses primary HPV testing as well as self‐sampling to broaden access to healthcare services [46]. The global use of self‐sampling is likely to increase in the coming years [45] and to ensure that the introduction does not exacerbate current inequalities in access to healthcare there is a need for timely communication about programme changes. SUCCEED is the first study to combine behaviour change and decision‐support theory to inform a communication strategy to facilitate informed decision‐making for mode of cervical screening. The study explored communication needs amongst individuals who are part of an organised cervical screening programme and findings could be relevant to other countries with similar programmes in preparation for communication plans introducing HPV self‐sampling as an option. In SUCCEED, we explored knowledge, values and preferences among cervical screening responders and those who have traditionally not taken up the screening invitation (including sub‐groups by age, socioeconomic group, ethnicity, gender identity and sexual orientation), as well as professional stakeholders about communication needs should HPV self‐sampling be incorporated into cervical screening programmes in the UK. We present two novel theory‐informed, evidence‐based logic models for a communication strategy with public health intervention targets for (1) communicating new cervical screening programme changes to the eligible population to raise awareness and promote equitable engagement and (2) supporting person‐centred informed decisions about self‐sampling and clinician‐sampling for HPV screening

In line with other recent UK and international studies [25, 47], participants were positive overall about the possibility of HPV self‐sampling. However some were sceptical about the reasoning behind the offer of a choice and cervical screening programme changes. This seems to be due to a perceived lack of transparency of the benefits of self‐sampling to both NHS staff and members of the public. Previous consumer‐based research has identified that scepticism can arise from a lack of transparency about reasons for introducing change [48]. Transparency about all outcomes, including those that are self‐serving, such as cost savings for national health institutions, have been shown in previous literature to reduce mistrust [48].

We found a lack of understanding about the overall aim of offering screening options, which is consistent with previous studies [31, 49]. Some participants felt they could opt into self‐sampling between other testing episodes undertaken by clinicians or if they developed relevant gynaecological symptoms. In addition to a general lack of understanding of screening as has been reported in other studies [50, 51, 52], we identified a low level of knowledge about HPV and cervical screening. There was misunderstanding of the current screening programme, which has for some nations shifted from cytology to primary HPV testing, meaning that the offer of HPV self‐sampling was incongruent with perceived current routine screening modes. This resulted in self‐sampling being seen as completely different, new and potentially inferior to clinician sampling. A novel finding from this study is the high perceived knowledge about cervical screening from the screening‐eligible population, despite this knowledge being factually inaccurate for many participants. Such misconceptions can influence the level of engagement that screening‐eligible people have with educational material. There is a need for a transparent whole‐population multi‐modal and multi‐agency (e.g. schools, public health, primary care, secondary care) campaign to promote understanding about why HPV self‐sampling is being introduced.

When discussing the offer of choice in cervical screening, decision‐support needs were high for participants. In line with other studies [30], concern over the accuracy of results and the potential cervical screening journey where there might be a need for clinician sampling in the case of HPV‐positive self‐sampling were raised. A clear need for multiplatform and multiformat tailored decision aids was identified. Core content focusing on choice introduction, clear justification for the need to offer self‐sampling, option presentation (comparison between clinician sampling and self‐sampling), and access to further support, like helplines or healthcare professional appointments, is required. Tailored content is needed for communities and individuals, such as those who have been through sexual violence, immigrants and certain religious and LGBTQ groups. A recent study has identified that individuals born outside the UK, adult immigrants and non‐English speakers sought stronger recommendations for cervical screening [53]. This highlights the need for culturally tailored interventions which explicitly present the reasons behind offering choice in cervical screening mode [54].

### 4.1 Strengths and Limitations

We applied a novel approach using theoretical integration to inform the development of a communication strategy for launching choice in cervical screening mode. We worked with TM to successfully recruit a diverse sample from a wide range of screening attendance, age and personal characteristics (including those not traditionally engaged in research). We also recruited a wide range of stakeholder roles. We worked closely with our public partners who helped guide the study and ensured that our work was relevant and accessible. They also helped analyse the qualitative data including suggestions for new themes. As with all qualitative work, the experiences described here are those of the people we spoke to and they may not be transferable to wider communities [55].

This study did not capture the views of screening‐eligible individuals or stakeholders from Northern Ireland (NI) or internationally. Primary HPV testing was only introduced in NI during this study (Dec 2023). Therefore, it will be important that perceptions from individuals from NI are incorporated in future work. The study was conducted in the UK, in the English language and excluded non‐English speakers, who are traditionally less likely to participate in cervical screening [56]. Further studies would benefit from a larger sample size informed by the PRICE model of data saturation [57] and recruiting from specific communities that might need tailored information, such as non‐English speaking individuals and those who have suffered sexual violence, to help further understand specific communication and decision‐support needs.

## 5 Conclusion

Self‐sampling in cervical screening could be an acceptable option to screening‐eligible individuals and stakeholders, so it could plausibly broaden screening participation. However, public misunderstanding of the motives for its introduction could lead to uninformed non‐participation. The introduction of HPV self‐sampling as a choice in countries with national cervical screening programmes needs to be carefully planned. Introduction needs to be supported by a robust lead‐in phase for a communication strategy to increase public awareness and engagement, accompanied by the development of multi‐modal decision support to facilitate informed decision**‐**making about cervical screening. The logic models presented in this study can inform the content of an overarching communication strategy to support informed, equitable participation.

## 6 Further Research

This study sets out the pathway for the creation of a communication campaign for supporting preference‐based cervical screening decisions. There is a need to develop and test the feasibility of the communication strategy informed by the logic models with coproduction from key community members who have been represented in this work and others who have not, such as non‐English speakers, individuals from other UK nations such as Northern Ireland as well as internationally and stakeholders from other sectors responsible for guiding health‐seeking behaviours in the population.

## Author Contributions

**Denitza Williams:** conceptualisation, investigation, funding acquisition, writing – original draft, methodology, validation, writing – review and editing, formal analysis, data curation, supervision, visualisation. **Eleanor Clarke:** investigation, writing – original draft, methodology, writing – review and editing, formal analysis, project administration, validation, data curation, software, visualisation. **Kate J. Lifford:** conceptualisation, funding acquisition, writing – review and editing, methodology. **Lindsay Haywood:** conceptualisation, funding acquisition, writing – review and editing, formal analysis, validation. **Fiona Wood:** conceptualisation, funding acquisition, writing – review and editing, methodology, supervision. **Jo Waller:** conceptualisation, funding acquisition, writing – review and editing, supervision, methodology. **Adrian Edwards:** conceptualisation, funding acquisition, writing – review and editing, methodology, supervision. **Natalie Joseph‐Williams:** funding acquisition, methodology, writing – review and editing, supervision. **Caroline Evans:** funding acquisition, conceptualisation, validation, formal analysis, writing – review and editing. **Gareth Powell:** funding acquisition, conceptualisation, supervision, writing – review and editing. **Rhiannon Phillips:** conceptualisation, funding acquisition, writing – review and editing, supervision. **Andrew Carson‐Stevens:** conceptualisation, funding acquisition, writing – review and editing, supervision. **Katie Walbeoff:** conceptualisation, funding acquisition, validation, writing – review and editing. **Ardiana Gjini:** conceptualisation, funding acquisition. **Kate Brain:** conceptualisation, funding acquisition, writing – review and editing, writing – original draft, methodology, validation, supervision.

## Ethics Statement

This study received approval from the School of Medicine Research Ethics Committee at Cardiff University Ref SMREC 22/88.

## Consent

All participants provided fully informed consent before taking part in the study. They were provided with a study information sheet and consent form and were given at least seven days between information provision and interview.

## Conflicts of Interest

The authors declare no conflicts of interest.

## Supporting information

Supporting information.

## Data Availability

All anonymised research data are available through the UK Data Service Repository (https://ukdataservice.ac.uk/about/).
