# Supplementary material for: Recommendations for a Communication Strategy to Support Informed Decision‐Making About Self or Clinician Sampling for Cervical Screening in the UK: Qualitative Study
Source: Health Expect. 2025 Mar 27;28(2):e70191. doi: 10.1111/hex.70191 (PMC11950154; doi:10.1111/hex.70191)
Supplement: Supplementary file 1 — Supporting information. [file HEX-28-e70191-s001.docx]

**Appendix 1 Interview Schedules for Screening eligible individuals and Stakeholders**

**Interview Schedule: Screening eligible individuals**

Study description

1. Interview prologue Introduce yourself, where you are calling from. Check they are happy to go ahead with an interview. Ask if they have read and understood the information sheet.
2. Set the focus of the interview/ Study description.

*At the moment cervical screening is* ***routinely offered*** *to individuals with a cervix between the ages of 25 and 64 years in the UK, it is sometimes called a smear test. In the future there could be a choice of how the screening test is done; either by a healthcare professional like a nurse in a clinic, like it is at the moment, or by the person themselves, which is called self-sampling. We want to understand what needs and preferences people might have about this choice of how to have the cervical screening test done. I’m talking to lots of people about this so we can get different points of view. I’m interested in what* ***you*** *think and so there are no right or wrong answers.*

Answer any questions.

*Does that make* ***sense****? Do have any questions about the study or about why I’m speaking to you today? Are you somewhere* ***private*** *as some of the things we discuss you may not want to talk about in public, or with people in earshot? I want you to feel comfortable and able to answer honestly, and I can call you back if there would be a better time or place for you.*

Consent discussion

Before we start I need to go through some paperwork with you to make sure you are happy and we do this properly for the study. I am going to read through a consent form and mark your answers. I need to record this and **I am turning the recorder on now.** Go through the consent form.

Now we have completed that I am going to switch that **recording off**, so that your details won’t be on the same file as our discussion.

1. Explain how the interview will work:

*I have some questions to guide our conversation today if that’s OK with you. If you find any question difficult to answer then just let me know and I can ask it in a different way. If you’d prefer not to answer any question, that’s fine, we can just move on, just let me know. If there’s anything that I don’t ask about that you think is relevant, please just tell me. Does that sound alright?*

*The interview may last about an hour. Please let me know if you need a break. Do you have enough battery on your device? Do you need to find a charger?*

1. Answer any further questions: *Do you have any* ***other questions*** *before we begin?*
2. I’m going to turn the **recorder on** now if you’re happy for us to start.

**Topics**

**A: Screening intentions**

*To start with I’d like to ask about whether you’ve previously taken part in cervical screening, which can be called a smear test, and reasons why you may or may not take part in the future.*

**1. Have you gone for cervical screening before?**

*Prompts: can you remember being* ***invited*** *(getting a letter)/ going for an appointment?*  ***Recent*** *attendance vs. Attended a long time ago.*

**2. When you’re next invited for cervical screening, do you think you’ll go?**

**3. Why do you think you will (not) go for cervical screening when you’re next invited?**

**(Or: If you are not sure, what do you feel will affect your decision?)**

*Prompts:*

*[Motivation]: Perceived* ***risk****, perceived* ***benefits****,* ***discomfort****, previous* ***experience****/results.*

*[Capability]:* ***Time****,* ***transport****,* ***disability****, ability to have screening (****self-efficacy****),* ***knowledge*** *and* ***understanding*** *of screening.*

*[Opportunity]:* ***invitation****,* ***convenience*** *(appointments, location).*

**B: Experience of communication in relation to cervical screening**

**4. How have you been invited to cervical screening in the past?**

*Prompts:* ***Letter*** *from GP/screening service, face-to-face invitation.*

**5. What information have you received or heard about cervical screening?**

*Prompts:* ***who*** *from (GP, nurse, mass media)? What* ***format*** *(leaflet, online, verbal)? Was it* ***useful****? Did it help you* ***decide*** *what to do? Do you feel like you* ***understood*** *enough about cervical screening? .*

**C: Attitudes towards choice in cervical screening**

*As I mentioned earlier, this study is exploring what people think about different ways to have cervical screening – it could be done in the usual way at a clinic by a healthcare professional, or in the future it might be possible for people to self-sample.*

**6. What do you think about potentially being offered a choice of cervical screening method in the future?**

Prompts: **Feelings** if offered choice, **who** should be offered choice, **timing** of offer [choice introduction].

**D: Decision support needs**

**7. What support do you think you might want with making a decision about which type of cervical screening if you were offered a choice in the future? [decision support tasks].**

*Prompts:* ***knowledge/information*** *about the options -* ***practicalities*** *of the test,* ***accuracy*** *of the test,* ***risks and benefits****,* ***decision aids****/booklets/discussions [decision support/evolving option presentation].*

***Talk*** *it through with anyone,* ***areas of sensitivity*** *that HCPs should be aware of [emotional support].*

*The test being developed for potential use in the future to carry out sampling for cervical screening at home is like a vaginal swab, so it is like a long cotton bud inserted by the person themselves to get a sample from the vagina. This is different to cervical screening carried out in a clinic where the health professional uses a speculum to open the vagina, to view and reach the cervix which is at the top of the vagina. This swab would then be sent to a lab for testing for HPV, and if there is no HPV then screening is repeated in 5 years and if there is HPV, then they would be referred for a cervical screening in clinic to get a sample from the cervix.*

**8. Now that I have explained this potential self-sampling how do you feel about this option?**

*Prompts: Does it make* ***sense****? What* ***else*** *do you think someone would need to know? Do you think it sounds* ***achievable****?*

**E: Communication preferences.**

**9. If a choice in screening is made part of the cervical screening programme, how would you like to hear about this?**

*Prompts e.g.* ***sources*** *of information (trusted sources),* ***type*** *of communication (media, letter from cervical* ***screening*** *service, letter from* ***GP****, text message etc)*

**10. How do you usually like to receive information about health? ? [evolving option presentation]**

*Prompts: e.g. if you have to make a decision about starting a new medication or going for a test.*

**11. How do you think you would potentially like to receive information specifically about a choice of cervical screening methods?**

*Prompts:* ***timing*** *(when brought in, before screening due),* ***who*** *(GP, nurse),* ***format*** *(face-to-face discussion, leaflet, online) [option presentation/planning discussion].*

**End of interview**

Thank the participant for their time.

Turn the **recorder off** and explain it is off

Answer any questions that may have arisen in the interview.

Ask if they’d like to receive a study summary in the future.

**Contacts for participants if needed**

**Cervical screening information**:

Wales: <https://phw.nhs.wales/>

England: https://www.nhs.uk/

Scotland: <https://www.nhsinform.scot/>

**Register with a GP**: instructions on NHS website

**Jo’s Trust**: Information and helpline on cervical screening, including some specifically for transgender men or non-binary people and easy to read instructions for those with a learning disability:

0808 802 8000; <https://www.jostrust.org.uk/>

**Cervical screening in women with vulval pain**: vulvalpainsociety.org: leaflet called ‘smears without tears’

**For people who have experienced sexual violence**: <https://mybodybackproject.com/> with specialist clinics and advice

**Interview schedule for Stakeholders**

Study description

1. Introduce yourself, where you are calling from.
2. Set the focus of the interview/ Study description.

*As you’re probably aware, at the moment cervical screening is routinely offered to individuals with a cervix between the ages of 25 and 64 years in the UK. Soon there could be a choice of how the screening test is done; either by a healthcare professional in a clinic, like it is at the moment, or by the individual themselves, which is called self-sampling. We want to explore how support for this decision could be done in practice.. I’m talking to lots of people about this so we can get different points of view. I’m interested in what you think and so there are no right or wrong answers.*

1. Check understanding and answer any questions.

*Does that make sense? Do have any questions about the study or about why I’m speaking to you today?*

Consent discussion

Go through the consent form (explain about audio-recording this if the interview is virtual)

Explain how the interview will work:

*I have some questions to guide our conversation today if that’s OK with you. If you find any question difficult to answer then just let me know and I can try to ask it in a different way. If you’d prefer not to answer any question, that’s fine, we can just move on, just let me know. If there’s anything that I don’t ask about that you think is relevant, please just tell me. Does that sound alright?*

*The interview may last about an hour. Please let me know if you need a break.*

*Do you have any other questions before we begin?*

1. I’m going to turn the recorder on now if you’re happy for us to start.

**Topics**

**Barriers and facilitators to the incorporation of HPV self-sampling**

*What do you think about including HPV self-sampling into the cervical screening programme?*

*Prompts: barriers/challenges & facilitators/enablers (communication of changes, practical, individual/participant, organisational, screening pathway).*

**Attitudes towards decision support and person centred care in cervical screening**

*What do you think about individuals’ choices about cervical screening?*

*Prompts: informed choice, discussions with eligible people about participation / shared decision making.*

*What kind of support do you think should be available for people making decisions about cervical screening?*

*Prompts: shared decision making/ discussions, information provision, patient decision aids.*

*How do you think a change in the cervical screening programme should be communicated to the public?*

*Prompts: timing, sources, mode of communication*

**Development of a combined behaviour change and decision support intervention**

*As I mentioned earlier, this study is aiming to develop an intervention to support the decision about cervical screening method - clinician vs. self-sampling.*

*What do you think about having a decision support intervention for the decision about screening method?*

*What do you think should be included in a decision support intervention?*

*How should the intervention be presented to Healthcare professionals?*

**Implementation challenges and facilitators of intervention**

*If a decision support intervention for method of screening was to be implemented into the cervical screening programme, what do you think the barriers and facilitators might be?*

*Prompts: practical, individual/participant or HCP, organisational, training needs, screening pathway.*

**End of interview**

Thank the participant for their time.

Answer any questions that may have arisen in the interview.

Ask if they’d like to receive a study summary.
